# Emergence and spread of a new community-genotype methicillin-resistant *Staphylococcus aureus* clone in Colombia

**DOI:** 10.1186/s12879-017-2193-3

**Published:** 2017-01-31

**Authors:** Javier Escobar-Perez, Niradiz Reyes, Ricaurte Alejandro Marquez-Ortiz, Juan Rebollo, Hernando Pinzón, Catalina Tovar, Jaime Moreno-Castañeda, Zayda Lorena Corredor, Betsy Esperanza Castro, Maria Victoria Moncada, Natasha Vanegas

**Affiliations:** 1 Bacterial Molecular Genetics Laboratory, Universidad El Bosque, Carrera 9 #131A-02, 110121274 Bogotá, DC Colombia; 2 Grupo de Genética y Biología Molecular, Universidad de Cartagena, Cartagena, Colombia; 3 Grupo de Investigación en Enfermedades Tropicales y Resistencia Bacteriana, Montería, Colombia; 4 Grupo de Microbiología, Instituto Nacional de Salud, Bogotá, Colombia; 5 I3 Institute, Faculty of Science, University of Technology, Sydney, Australia

**Keywords:** Methicillin-resistant *Staphylococcus aureus*, Community, New clone, Colombia

## Abstract

**Background:**

Community-genotype methicillin-resistant *Staphylococcus aureus* (CG-MRSA) clones are a global concern due to their resistance and increased virulence and their ability to cause infections both hospitalized patients and healthy people in the community. Here, we characterize 32 isolates of a new CG-MRSA clone. These isolates were identified in four cities in Colombia, South America.

**Methods:**

The isolates were recovered from four different epidemiological and prospective studies that were conducted in several regions of Colombia. Molecular characterizations included multilocus sequence typing; pulsed-field gel electrophoresis; SCC*mec, agr* and *spa* typing; and whole-genome sequencing.

**Results:**

All isolates belonged to ST923 (clonal complex 8), harbouring SCC*mec* IVa and a *spa* type t1635 and lacking an arginine catabolism mobile element. The isolates were classified as COL923, were resistant to at least one non-beta-lactam antibiotic, and exhibited high frequencies (>60%) of resistance to macrolides and tetracycline. Using whole-genome sequencing, we found that this new clone harbours novel prophage 3 and beta-island structures and a slightly different pathogenicity island 5. Moreover, isolates belonging to the COL923 clone are grouped in a different clade than USA300 and USA300-LV.

**Conclusion:**

Our results show the emergence and spread of the COL923 clone in different cities in Colombia. This clone is resistant to several antibiotics and possesses new structures in its mobile genetic elements.

**Electronic supplementary material:**

The online version of this article (doi:10.1186/s12879-017-2193-3) contains supplementary material, which is available to authorized users.

## Background

The emergence, dissemination and establishment of community-genotype methicillin-resistant *Staphylococcus aureus* (CG-MRSA) clones is a global concern due to their increased virulence and their enhanced ability to cause infections in healthy people compared with hospital-genotype MRSA (HG-MRSA) clones. In addition, the frequency of CG-MRSA clones has increased among MRSA infections in hospitalized patients in several countries worldwide [1–4], which implies the replacement of traditional HG-MRSA clones by CG-MRSA clones in the hospital setting and also demonstrates acquisition of resistance to non-beta-lactam antibiotics. USA300 is a CG-MRSA pandemic clone that is circulating in North America [1] but has also been reported on every other continent, including in countries such as Denmark, Spain, France, Italy, Norway, Austria, the United Kingdom, Germany, Japan, Korea, Singapore and Australia [5–8]. This distribution of this clone demonstrates its ability to spread. Since 2004, a CG-MRSA clone that is genetically related to USA300 has been found to cause infections in adults and children in Colombia and several other South American countries [6, 9, 10]. In contrast to USA300, this variant harbours SCC*mec* IVc (3.1.2) element but lacks an arginine catabolism mobile element (ACME). In recent years, the frequency of this MRSA-IVc clone has increased among MRSA infections both in the community and in hospitalized patients, replacing traditional HG-MRSA clones (e.g., Chilean and Brazilian clones) [11, 12]. We recently reported the detection of eight isolates belonging to a new CG-MRSA clone that have caused paediatric infections in Bogota, Colombia [13]. These isolates were found to possess SCC*mec* IVa and a *spa* type t1635; to belonged to the ST923 (a single-locus variant of ST8); to possess an *Sma*I-restriction pulsed-field gel electrophoresis (PFGE) pulsotype that is not related to that of the USA300 clone (>6 bands of difference); and to lack an ACME, which is frequently found in the USA300 clone. To determinate whether this new clone is circulating in other areas of Colombia, we conducted an active search for this clone and performed a genomic characterization. In this study, we show that this new CG-MRSA clone (named COL923 by our group) circulates in at least four regions of Colombia and possesses new clone-specific molecular characteristics.

## Methods

### Bacterial isolate collection and identification

A total of 32 CG-MRSA isolates (containing SCC*mec* type IV and/or possessing the gene *lukS/F-PV*, *seq*, *sek* or *bsaB*) belonging to the new clone COL923 (SCC*mec* IVa, *spa* type t1635 and ST923) were identified in four different studies (Additional file 1: Table S1). In the first study, 430 *S. aureus* isolates were prospectively and systematically recovered from infections in paediatric patients (<18 years old) at a hospital in Cartagena, which is a city on the Caribbean coast. Nineteen of the MRSA isolates were related to COL923 (one isolate per patient). In the second study, MRSA nasal colonization was investigated in 2867 healthy adult individuals (>18 years old) living in 10 regions that are geographically distant from Colombia. Of the isolates obtained, three isolates from two different regions were related to COL923. In the third study, MRSA nasal colonization was investigated in 150 healthy children (<5 years old) in Monteria, which is a city in northwest Colombia. Of the isolates obtained, six isolates were related to COL923. Finally, in the fourth study, MRSA nasal colonization was evaluated in 205 healthy children (5 to 14 years old) in Cartagena, and four CG-MRSA isolates were found to belong to the new clone.

### Antimicrobial susceptibility testing

The profile of susceptibility to 12 antibiotics (oxacillin, gentamicin, rifampicin, erythromycin, ciprofloxacin, vancomycin, linezolid, tetracycline, clindamycin, chloramphenicol, trimethoprim and sulfamethoxazole) was determined for each of the isolates using the agar dilution method. The results were interpreted according to the 2015 guidelines of the Clinical and Laboratory Standards Institute (CLSI). Inducible clindamycin resistance was also determined using the D-test.

### Molecular characterization and establishment of genetic relatedness of isolates

The SCC*mec* type and subtype were established for each clone using multiplex PCR, as previously described [14]. The *lukS/F-PV*, *etb*, *eta*, *hlg*, *sea*, *seb*, *sec*, *seg*, *seh*, *sei*, *sej*, *sek*, *sel*, *sem*, *sen*, *seo*, *sep* and *seq* genes were also analysed using PCR [15]. In addition, the presence of the *blaZ, tetK, tetM, ermA, ermB, ermC, msrA, mph* and *mefA* genes was evaluated in all isolates. Genetic relatedness between the isolates was determined by PFGE. The obtained pulsotypes were interpreted according to the percentage of similarity and the criteria proposed by Tenover and colleagues [16]. The *agr* group, *spa* type and multilocus sequence typing were determined as previously reported [17].

### Detection of mobile genetic elements (MGEs) and genome sequencing of new CG-MRSA clone

**Fig. 1:**
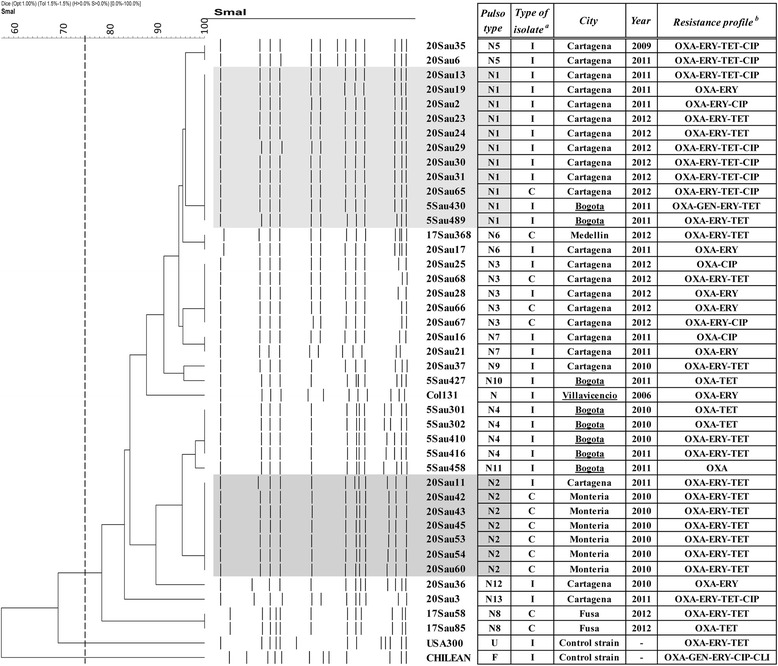
Genetic relatedness among isolates belonged to the COL923 clone identified in Colombia. The isolates underlined correspond to the isolates identified previously. USA300-0114-FPR3757 and CHL93 (Chilean clone) strains were used as standards. PFGE patterns were considered different when they showed a similarity lower than 75% (*broken line*). Shaded region indicates the two more frequent pulsotypes. *a*. Type of isolate: I = infection, C = Colonization. *b*. OXA = oxacillin, TET = tetracycline, ERY = erythromycin, GEN = gentamicin, CIP = ciprofloxacin, CLI = clindamycin

The presence or absence of the most important MGEs and genomic islands (GIs) was evaluated using different PCR strategies (Additional file 2: Figure S1). For the genome sequencing analysis, isolates were selected as follows: for the COL923 clone, two representative isolates were selected for each of the two main PFGE pulsotypes (isolates 5sau489 and 17sau368, belonging to the first PFGE pulsotype (Fig. 1), and isolates 5sau410 and 17sau58, belonging to the second PFGE pulsotype (Fig. 1)). In addition, Col131 was selected because it was the first isolate related to the COL923 clone that was identified in Colombia. For the USA300-LV clone (the most frequent CG-MRSA clone in Colombia), one representative isolate was selected for each of the four most frequent PFGE pulsotypes (i.e., 17sau599, 5sau003/17sau366, 17sau193 and 17sau391). Total DNA was extracted from the 10 MRSA clinical isolates using the PureLink® Genomic DNA Mini Kit from Thermo Fisher. The DNA was used to prepare multiplexed total DNA libraries using the Nextera XT Dual Index Sequencing Primer Kit (Illumina, Inc.) and the KAPA Library Amplification Kit (Kapa Biosystems). The multiplexed libraries were pooled and sequenced by paired-end sequencing using the Illumina MiSeq (2x300 cycles) and HiSeq (2x101 cycles) platforms. The libraries were assembled using SOAPdenovo2 V2.04-r240, with kmer values of 63 and 127 for HiSeq and MiSeq, respectively. The basic assembly statistics are shown in Additional file 3: Table S2. The reads were also mapped to the USA300-FPR3757 genome using SHRiMP 2.2.3 and were then compared and visualized using BRIG software [18, 19]. The genetic relatedness of the sequenced isolates was established using phylogenetic analysis. To build a phylogenetic tree, the partially assembled (i.e., 17sau193, 17sau366, 17sau368, 17sau391, 17sau58, 17sau599, 5sau003, 5sau410, 5sau489 and Col131) and reference (i.e., USA300 and NCTC8325) genomes were annotated using Prokka [20] and an alignment was created for 2288 concatenated core genes (genes with ≥99% nucleotide identity that were present in all genomes) using Roary [21] and PRANK [22]. Poorly aligned positions and divergent regions were eliminated using Gblocks [23]. Finally, the phylogenetic tree was created using RAxML version 8.2.9 [24] by running 1000 bootstrap replicates under the generalized time-reversible model (GTRCAT). Finally, a consensus tree was plotted using Dendroscope [25]. Branch lengths are expressed in units of changes/nucleotide position (scale bar). The NCTC8325 strain was included as an outgroup control (GenBank ID: NC_007795.1). Different MGEs were identified from the mapping analysis and then annotated using the RAST server [26].

## Results

Among the 32 CG-MRSA isolates belonging to the COL923 clone that were analysed, 19 were recovered from paediatric infections, and 13 were recovered from nasal samples from healthy youths and children. Among the 19 infection isolates, one was identified in 2009; two, in 2010; nine, in 2011; and seven, in 2012. The main clinical diagnosis was skin or soft-tissue infection (10 cases). Four patients developed septic shock, and of these, one also developed pneumonia (Table 1). Eighteen patients were definitively treated for MRSA infections with active antimicrobials, and one was treated with oxacillin. Thirteen patients (68.4%) were additionally managed with incision and drainage with full recovery observed in all 13 cases. Four patients (21.05%) required a paediatric intensive care unit stay because of the severity of their infections. All (100%) infections were identified within the first 48 h of hospital admission in children without healthcare-associated risk factors who entered the hospital through the emergency department (i.e., community-onset MRSA infections).

**Table 1:** Demographic and clinical characteristics of paediatric patients with MRSA infections

Demographic data and clinical characteristics	All subjects (*n* = 19)
	n (%)
Age (years) (range)	8.3 (0.17–14)
Age distribution	
Newborn	0 (0.0)
Infants	1 (5.3)
Pre-school age	4 (21.1)
School age	7 (36.8)
Adolescents	7 (36.8)
Male sex, %	12 (63.0)
Year of recovery (time in months)^a^	
2009 (3)	1 (5.3)
2010 (12)	2 (10.5)
2011 (12)	9 (47.4)
2012 (6)	7 (36.8)
Admission Site	
Emergency room	19 (100.0)
Clinical diagnosis	
SSTI^b^	10 (43.8)
Osteoarticular infection	5 (26.0)
Pneumonia	1 (5.6)
Septic Shock	3 (2.5)
Onset time of symptoms (mean, SD^c^), days	17.4 (20.0)
Hospital management^d^	
Hospitalized- drainage	15 (69.1)
PICU	4 (24.1)
Empirical antimicrobial therapy^e^	
CLI	13 (68.4)
OXA	2 (10.5)
CLI/RIF	2 (10.5)
RIF	1 (5.3)
CLI/RIF/VAN	1 (5.3)
Definitive antimicrobial therapy^e^	
SXT	11 (57.9)
CLI/RIF	3 (15.8)
OXA	1 (5.3)
RIF	1 (5.3)
CLI	1 (5.3)
CLI/RIF/VAN	1 (5.3)
VAN/LZD/SXT	1 (5.3)
Clinical outcome	
Improvement	19 (100.0)

^a^The collection time of the isolates was between October 2009 and June 2012

^b^ *SSTI* Skin and soft tissue infection

^c^ *SD* standard deviation

^d^ *PICU* paediatric intensive care unit

^e^ *CLI* clindamycin, *OXA* oxacillin, *RIF* rifampicin, *SXT* trimethoprim-sulfamethoxazole, *VAN* vancomycin, *LZD* linezolid

In contrast, the 13 MRSA nasal colonization isolates were identified and recovered from healthy people living in Medellin, Cartagena, Monteria and Fusagasuga, which are four cities both distant from each other and distant from Bogota and Villavicencio, where the COL923 strain was first reported. The fact that we did not find any epidemiological link among the people harbouring this clone suggested that this CG-MRSA clone was already circulating in several regions of the country.

### Antimicrobial resistance profiles and resistance mechanisms

All isolates were susceptible to vancomycin, linezolid, clindamycin, gentamicin, rifampicin, chloramphenicol and trimethoprim/sulfamethoxazole. However, the minimal inhibitory concentration (MIC) for vancomycin was 1 mg/L. Of the 32 COL923 isolates, 30 (93.8%), 21 (65.6%) and 12 (37.5%) were resistant to erythromycin, tetracycline and ciprofloxacin, respectively (Table 2). Additionally, all isolates were resistant to at least one non-beta-lactam antibiotic. The most frequent multiresistance profiles consisted of erythromycin-tetracycline resistance (40.6%) or erythromycin-tetracycline-ciprofloxacin resistance (25.0%) (Table 2). The *blaZ* gene was detected in all of the isolates. All erythromycin-resistant isolates (30) presented an M phenotype and harboured the *msrA* and *mphC* genes. The 21 isolates that were resistant to tetracycline harboured the *tetK* gene. The *blaZ, tetK, msrA* and *mphC* genes were localized in contigs that did not map to the USA300-FPR3757 genome, suggesting the possibility of transmission by plasmids, although this needs to be confirmed in future work.

**Table 2:** Antimicrobial resistance and molecular characteristics of MRSA isolates belonging to the COL923 clone

Characteristics of isolates	All isolates (*n* = 32)	Infection (*n* = 19)	Colonization (*n* = 13)
	n (%)	n (%)	n (%)
Antimicrobial resistance^a^			
ERY	30 (93.8)	17 (89.5)	13 (100.0)
TET	21 (65.6)	11 (57.9)	10 (76.9)
CIP	12 (37.5)	10 (52.6)	2 (15.4)
Multiple antibiotics resistance profiles			
OXA-ERY-TET	13 (40.6)	4 (21.1)	9 (69.2)
OXA-ERY-TET-CIP	8 (25.0)	7 (36.8)	1 (7.7)
OXA-ERY	7 (21.9)	5 (26.3)	2 (15.4)
OXA-CIP	2 (6.3)	2 (10.5)	0 (0.0)
OXA-ERY-CIP	2 (6.3)	1 (5.3)	1 (7.7)
Only OXA^b^	0 (0.0)	0 (0.0)	0 (0.0)
Main PFGE pulsotype^c^			
N1	9 (28.1)	8 (42.1)	1 (7.7)
N2	7 (21.9)	1 (5.3)	6 (46.2)
N3	5 (15.6)	2 (10.5)	3 (23.1)
N5	2 (6.3)	2 (10.5)	0 (0.0)
N6	2 (6.3)	1 (5.3)	1 (7.7)

^a^ *OXA* oxacillin, *TET* tetracycline, *ERY* erythromycin, *GEN* gentamicin, *CIP* ciprofloxacin, *CLI* clindamycin

^b^Susceptibility to non-B-lactam antibiotics

^c^N4 pulsotype was assigned to isolates recovered from Bogota (Fig. 1)

### Molecular characteristics and genetic relatedness of isolates

The molecular characterization of the 32 CG-MRSA isolates demonstrated that they all harboured SCC*mec* IVa (IV.1.1.1), were of *spa* type t1635 (YHGFMBO) and presented a 54-bp insertion in the *sausa300_0808* gene in *S. aureus* pathogenicity island 5 (SaPI5). These same features were previously found in the COL923 isolates recovered from paediatric infections in Bogota, Colombia [13]. All isolates harboured the *sek* and *seq* genes within SaPI5. The *lukS/F-PV* genes were detected in 29 (90.6%) isolates. An ACME was not detected in any of the isolates. The PFGE analysis of the 32 isolates revealed the presence of 10 different pulsotypes (the N1 to N3, N5 to N9, N12 and N13 pulsotypes, as shown in Fig. 1). The N1 and N2 pulsotypes were the most frequent, found in 9 (28.1%) and 7 (21.9%) isolates, respectively (Table 2). All 32 isolates included in this study showed similarities greater than 80% with respect to the isolates previously recovered in Bogota [12] (Fig. 1). Finally, all isolates belonged to *agr* group I and the sequence type 923.

### Detection of MGEs, GIs and genome comparison

**Fig. 2:**
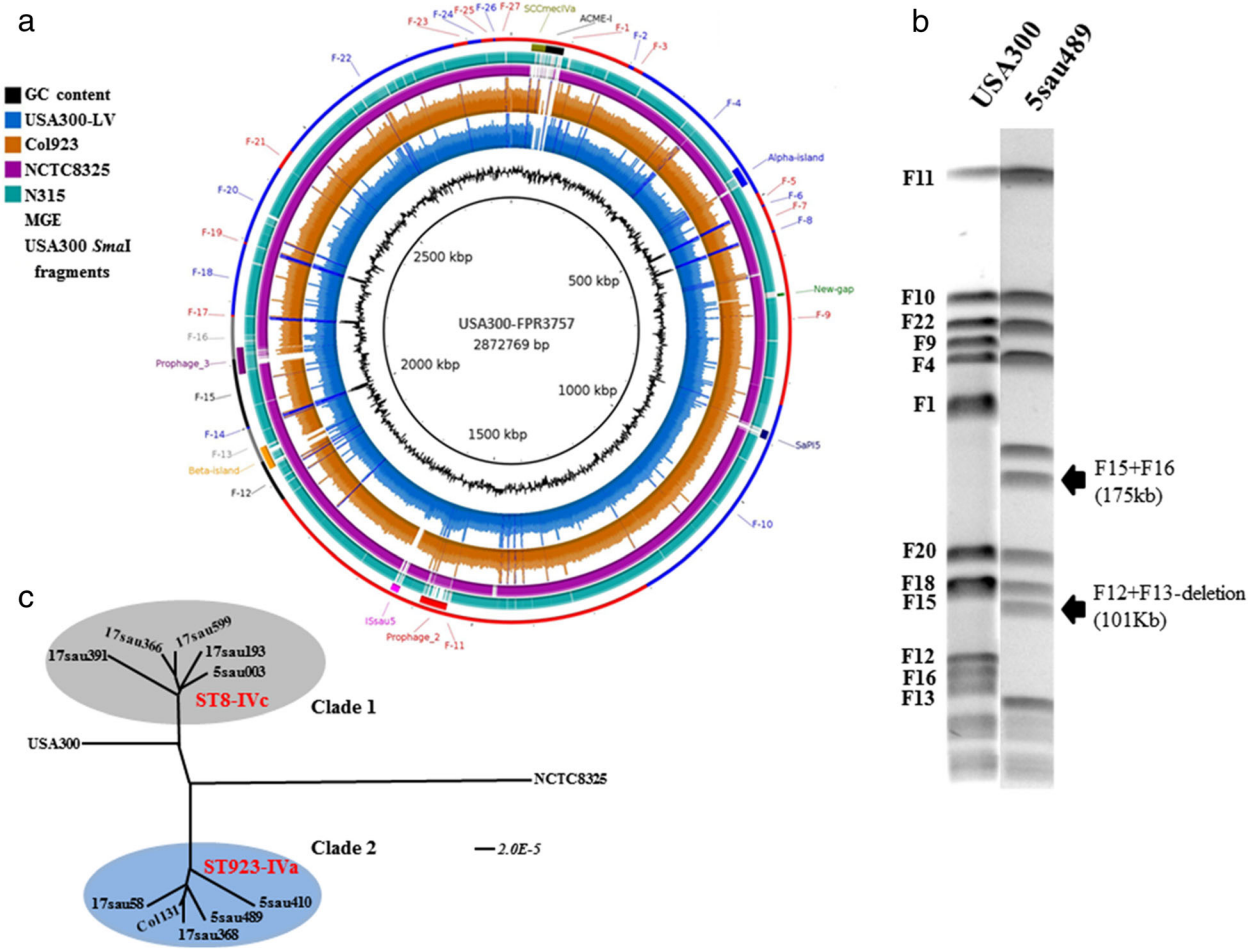
Genomic comparison of COL923, USA300-LV and USA300-FPR3757 clones. **a** Read mapping coverage of sequencing reads of COL923 clone (5sau489 isolate as representative for the most frequent pulsotype, *blue circle*) and USA300-LV (5sau003, *orange circle*), compared to the USA300-FPR3757 genome (GenBank number: CP000255.1), the *black most inner ring*. The *outer circle* (*alternating red-blue*) represents the USA300 *Sma*I fragments (responsible for the PFGE pulsotype). *Black and grey fragments* represent fragments that change in the COL923 clone, by loss of the *Sma*I recognition sites. Matches with less than 80% of nucleotide identity or regions without match to the reference genome appear as blank in each ring. The image was prepared using BRIG. **b** *Sma*I-restriction PFGE pulsotypes of USA300 and COL923 (5sau489 isolate) clones. *Black arrows* show the two new fragments in the COL923 clone which were produced for the loss of the *Sma*I cuts between the fragments 12 and 13, and fragments 15 and 16 (*black and grey fragments in figure a*). **c** Maximun likelihood phylogenetic analysis using core genomes from representative isolates belonging to the COL923 clone (clade 2) and USA300-LV (clade 1). The USA300 clone grouped in a different clade more related to the USA300-LV

**Fig. 3:**
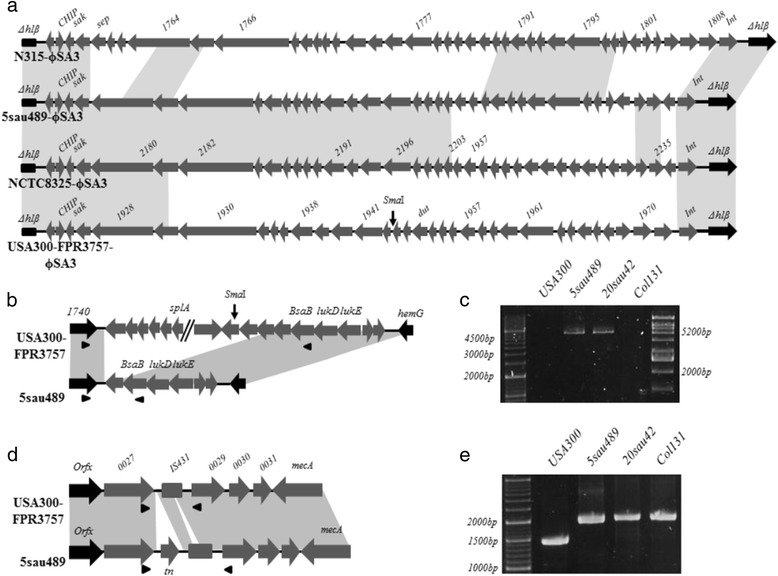
Novel structures in the mobile genetic elements in the COL923 clone. Schematic diagrams of the prophage 3, Beta Island and SCC*mec* J3 region. **a** Prophage 3 structures identified in COL923 (5sau489), N315 (GenBank number: BA000018.3), NCTC8325 (GenBank number: CP000253.1) and USA300-FPR3757 (GenBank number: CP000255.1). **b** Beta island structures identified in COL923 (5sau489) and USA300-FPR3757 (GenBank number: CP000255.1). The beta island size in COL923 (5sau489) was 13,980bp. **c** PCR amplification of a partial region of the beta island in COL923 (5sau489, 20sau42 and Col131 isolates) and USA300-FPR3757. **d** Structure of the SCC*mec* J3 regions identified in COL923 (5sau489) and USA300-FPR3757. **e** PCR amplification of the partial J3 SCC*mec* region that contains the putative ORF encoding for a transposase (tn) in COL923 (5sau489, 20sau42 and Col131 isolates). Shaded regions represent sequences with >95% of nucleotide identity. *Sma*I: restriction site for the *Sma*I enzyme. *Black arrows* represent the ORFs localized upstream and downstream of the mobile genetic elements and genomic islands. Primers used in PCR amplifications are showed as *black triangles*

The assembly statistics for the sequenced genomes are shown in Additional file 3: Table S2. The genomic mapping of the COL923 clone revealed certain differences with respect to the USA300-FPR3757 and USA300-LV clones (Fig. 2). These differences were mainly due to changes in the MGEs (e.g., changes in the prophage 3, beta-island, and SCC*mec* J3 regions and the absence of ISsau5) in addition to single-nucleotide polymorphisms in the core genome. It is also important to highlight that the beta-island (*υ*Saβ) was smaller in the COL923 clone (13,980 bp) than in the USA300-FPR3757 and USA300-LV (37,590 bp) clones, displaying a truncated structure. PCR analysis showed that all isolates (except Col131) possessed this truncated beta-island (*υ*Saβ) structure (Fig. 2a and Fig. 3b and c). All isolates harboured the *sak* gene (a marker of prophage 3), and genome sequencing confirmed the presence of prophage 3 (ϕSA3) but indicated that it possessed a new genetic structure that best matched the NCTC8325 strain (63.4% identity) (Fig. 3a). However, this prophage 3 (ϕSA3) shared low sequence identity with the USA300-FPR3757 (38.4%) and N315 (39.6%) prophage 3 (ϕSA3). Additionally, the genome sequencing analysis of 5sau489 revealed two DNA insertions in the SCC*mec* J3 region that were not present in USA300-FPR3757 (Fig. 3d). The first insertion (578 bp) harboured an open reading frame (ORF) that encoded a putative transposase, which had previously been reported in another CG-MRSA-ST72-IVa strain that was identified in South Korea [27]. PCR analysis showed that all isolates (including the 32 isolates described in this study and 9 isolates identified previously) contained two DNA insertions in the J3 region of SCC*mec* (Fig. 3e). These findings show that the COL923 clone possesses SCC*mec* IVa that is not identical to that in USA300.

The genetic structure of νSaα in the isolates described in this study was the same as that reported for the USA300 clone [28]. A maximum-likelihood phylogenetic analysis of the core genomes showed that the isolates could be segregated into two distinct clades (Fig. 2c). Clade 1 consisted of isolates belonging to the USA300-LV clone (ST8-IVc), while clade 2 consisted of isolates belonging to the COL923 clone (ST923-IVa). The USA300 clone was not part of any clade; nevertheless, it was more closely related to the isolates in the USA300-LV clade. Taken together, these data strengthen the idea that the COL923 clone is genetically different with respect to the USA300 and USA300-LV clones, in addition to the differences observed in MGEs.

## Discussion

The extraordinary genetic success of CG-MRSA clones is now well known, and we have observed that its frequency has increased during the last two decades, first in the community and then in the hospital setting, in several countries around the world. In Colombia, several genetic and molecular analyses have shown that the most prevalent CG-MRSA clone (USA300-LV) possesses certain characteristics that are similar to those of the USA300 pandemic clone. However, the Colombian CG-MRSA clone harbours a different SCC*mec* (IVc or IV.3.1.2) and, interestingly, does not possess an ACME [6, 11, 29–31]. Between 2008 and 2011, the prevalence of this clone was reported to comprise 70% and 90% of MRSA infections in adults and paediatric patients, respectively [6, 11, 30], in several Colombian cities. However, in 2010, we identified eight CG-MRSA isolates with different molecular and genetic characteristics compared to the USA300-LV clone but with similarity to the COL923 clone [12, 13]. Our data showed that the COL923 (CG-MRSA-IVa-ST923) clone had also been causing infections in children in other regions of Colombia (outside Bogota) since 2009. This new clone was identified in five distant geographic areas, which suggests that it was already circulating in several regions of our country and that its emergence represents a dissemination event, and not an epidemic event in one city [11, 29, 32].

In addition, whole-genome sequencing analysis revealed unique characteristics in both the MGE and the core genome of this new CG-MRSA clone. With respect to SCC*mec*, certain variations were found in its J3 region (Figs. 2 and 3d). For example, a 578-bp insertion was identified that has also been recently reported in SCC*mec* IVa element harboured in an ST72 (CC8, triple-locus variant of ST8) clinical isolate that caused a community-onset infection [27]. This particular region contains an ORF that encodes a putative transposase. Although the SCC*mec* IVa element in the COL923 clone still has high nucleotide identity (>98%) with the SCC*mec* IVa element in the USA300 clone, our results show that it has also acquired certain foreign DNA fragments [12, 13]. These results reinforce the idea that the new variant (COL923) has gained DNA fragments and support the importance of further studies aimed at increasing our understanding of this process.

The novel results of the present study include the identification of new prophage 3 (ϕSA3) and beta-island (*υ*Saβ) structures in the COL923 clone. To our knowledge, these structures have not been previously reported. According to these results, the new prophage 3 has low genetic relatedness to the prophage 3 that was previously identified in the USA300 clone. Based on its structure and size, this new prophage can be classified into the Siphoviridae family, and it combines parts of other prophages into a mosaic structure. Thus, we could hypothesize that it originated during a series of events that involved recombination among the functional modules of different prophage 3 types, a process that has been previously reported in Siphoviridae prophages [33]. The insertion of this new prophage 3 and a truncated beta-island caused the loss of two *Sma*I restriction sites in the chromosome of the new clone, leading to a change in the PFGE pulsotype (Fig. 2).

In 2006, Alvarez et al. reported recovering CG-MRSA isolates (Col131) from an infection in an adult patient in 2004 [9, 34], Subsequent molecular and genetic analyses showed that this Col131 isolate harboured SCC*mec* IVa. The molecular analysis of the Col131 isolate that we performed showed that it shared molecular characteristics with the new COL923 clone (ST923, *spa* type t1635, *sausa300_0808*-variant gene, prophage 3, SaPI5 and J3-SCC*mec* structure) but had a different PFGE pulsotype (Fig. 1). Interestingly, since 2004, the USA300-LV clone has shown increasing frequency in MRSA infections, whereas the COL923 clone has shown a very low frequency. These findings suggest two possible hypotheses. First, the Col131 isolate, after acquiring SCC*mec* IVa, may have undergone several genetic changes that resulted in environmental advantages that gave rise to the COL923 isolates identified since 2009. Second, certain ST923 methicillin-sensitive *S. aureus* isolates with similar molecular characteristics and genetic relatedness to the Col131 isolate may have acquired the same SCC*mec* IVa during different events.

It is important to highlight that among the COL923 isolates identified here, four were resistant to the non-beta-lactam antibiotics ciprofloxacin, erythromycin and tetracycline. The last two types of resistance are associated with the *msrA, mphC* and *tetK* genes, which are possibly carried by plasmids (these were not mapped in the reference chromosomes), suggesting that plasmids are mobilizing among CG-MRSA clones present within the community in Colombia. We hypothesize that both selective pressure due to the overuse of antibiotics and the unnatural accumulation of certain substances in the community have filtered the local *S. aureus* population to favour the new variant, which likely has better fitness because of its *msrA-* or *tetK*-positive plasmid. In comparison with the USA300 and USA300-LV clones circulating in our region, the COL923 clone displays a broader resistance range and an MIC to vancomycin of 1 mg/L, even outside the hospital setting. The rise in the frequency of this clone is therefore a possible challenge to the health system.

## Conclusion

Our data demonstrate that a new CG-MRSA COL923 clone is circulating in different regions of Colombia, rather than being confined to a single city. Additionally, this clone possesses new genetic structures that have not been previously reported that differentiate it from the USA300-LV and USA300 clones. The COL923 clone is a new CG-MRSA clone that is causing infections in people in the community and that is also having an impact within the health system. It has been frequently observed that although many clones possess the ability to acquire the same SCC*mec*, only a few prevail, with certain clones predominating in their respective geographic niches [35]. This finding suggests that specific genetic determinants govern the predominance of different clones in different regions of the globe. Thus, it is necessary to continue surveillance studies in both the community and hospitals to assess the clinical, economic and social impacts of the dissemination of this new CG-MRSA clone.

## Additional files

Additional file 1 Table S1. Additional file 2: Figure S1 Schematic diagram used to detect the main Mobile Genetic Elements (EMG) in the MRSA isolates. The genome sequence reported to USA300-FPR3757 (GenBank accession number CP000255.1), COL (GenBank accession number CP000046.1), Mu50 (GenBank accession number BA000017.4) and N315 (GenBank accession number BA000018.3) were used as reference. Black arrows represent the ORFs localized upstream and downstream of the EGM and GI. Black triangles represent the PCR primers localization. Abbreviations: SaPI: *Staphylococcus aureus* Pathogenicity Island, ϕSA: *S. aureus* Prophage, νSaα: *S. aureus* genomic island Alfa, νSaβ: *S. aureus* genomic island beta and νSa4: *S. aureus* genomic island 4. (TIF 608 kb)
Additional file 3: Table S2 Assembly statistics to 10 MRSA isolates sequenced using the MiSeq platform (except 5sau003 and 5sau489, sequenced using HiSeq 2000). L50 and L75 are the number of contigs greater than N50 and N70 respectively. (DOCX 15 kb)

## Abbreviations

ACME: Arginine catabolic mobile element
*agr*: Accessory gene regulator
CG-MRSA: Community-genotype methicillin-resistant *Staphylococcus aureus*
CLSI: Clinical and Laboratory Standards Institute
GI: Genomic islands
HG-MRSA: Hospital-genotype methicillin-resistant *Staphylococcus aureus*
MGE: Mobile genetics elements
MLST: Multilocus sequence typing
MSSA: Methicillin-sensible *Staphylococcus aureus*
ORF: Open reading frame
PCR: Polymerase chain reaction
PFGE: Pulsed-field gel electrophoresis
SaPI5: *Staphylococcus aureus* pathogenicity island 5
SCC*mec*: Staphylococcal cassette chromosome mec
*spa*: Staphylococcal protein A
ST: Sequence type
UPGMA: Unweighted Pair Group Method with Arithmetic mean
USA300-LV: USA300-latinAmerican variant
WGS: Whole-genome sequencing
