# Supplementary figures and images for: Emergence and spread of a new community-genotype methicillin-resistant Staphylococcus aureus clone in Colombia
Source: BMC Infect Dis. 2017 Jan 31;17:108. doi: 10.1186/s12879-017-2193-3 (PMC5282769; doi:10.1186/s12879-017-2193-3)

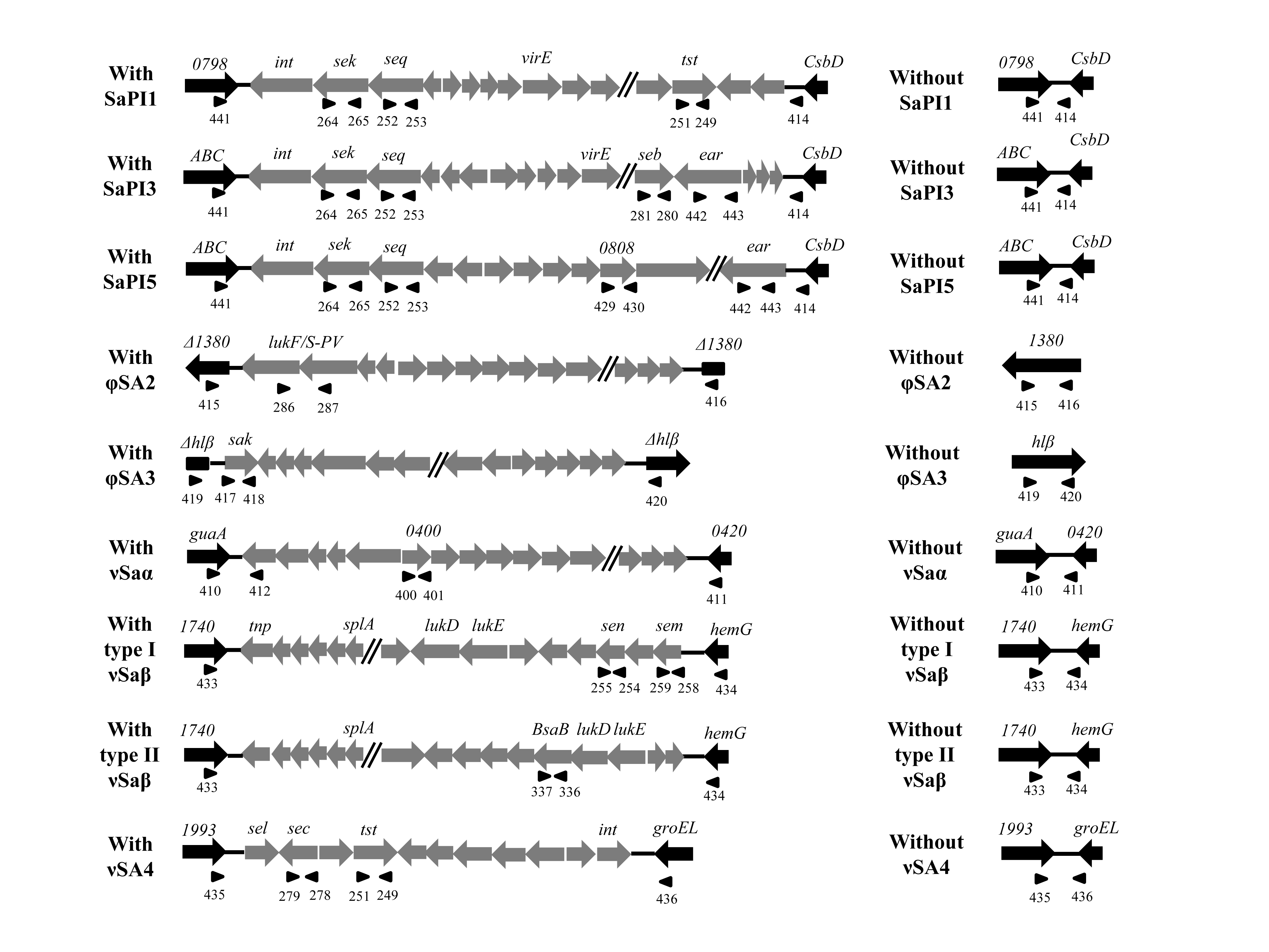

Supplement: Additional file 2: Figure S1 — Schematic diagram used to detect the main Mobile Genetic Elements (EMG) in the MRSA isolates. The genome sequence reported to USA300-FPR3757 (GenBank accession number CP000255.1), COL (GenBank accession number CP000046.1), Mu50 (GenBank accession number BA000017.4) and N315 (GenBank accession number BA000018.3) were used as reference. Black arrows represent the ORFs localized upstream and downstream of the EGM and GI. Black triangles represent the PCR primers localization. Abbreviations: SaPI: Staphylococcus aureus Pathogenicity Island, ϕSA: S. aureus Prophage, νSaα: S. aureus genomic island Alfa, νSaβ: S. aureus genomic island beta and νSa4: S. aureus genomic island 4. (TIF 608 kb) [file 12879_2017_2193_MOESM2_ESM.tif]
