# Supplementary material for: Emergence and spread of a new community-genotype methicillin-resistant Staphylococcus aureus clone in Colombia
Source: BMC Infect Dis. 2017 Jan 31;17:108. doi: 10.1186/s12879-017-2193-3 (PMC5282769; doi:10.1186/s12879-017-2193-3)
Supplement: Additional file 3: Table S2 — Assembly statistics to 10 MRSA isolates sequenced using the MiSeq platform (except 5sau003 and 5sau489, sequenced using HiSeq 2000). L50 and L75 are the number of contigs greater than N50 and N70 respectively. (DOCX 15 kb) [file 12879_2017_2193_MOESM3_ESM.docx]

**Table S2.**  Assembly statistics to 10 MRSA isolates sequenced using the MiSeq platform (except 5sau003 and 5sau489, sequenced using HiSeq 2000). L50 and L75 are the number of contigs greater than N50 and N70 respectively.

| Assembly | **17sau193** | **17sau366** | **17sau368** | **17sau391** | **17sau58** | **17sau599** | **5sau003** | **5sau410** | **5sau489** | **Col131** |
| --- | --- | --- | --- | --- | --- | --- | --- | --- | --- | --- |
| Average coverage depth | 395 | 359 | 395 | 416 | 441 | 478 | 152 | 439 | 157 | 455 |
| # contigs (≥0 bp) | 82 | 107 | 122 | 67 | 97 | 106 | 75 | 106 | 97 | 51 |
| # contigs (≥5000 bp) | 46 | 39 | 50 | 39 | 43 | 61 | 41 | 52 | 46 | 33 |
| Total length (≥0 bp) | 2904349 | 2983298 | 2927009 | 2794859 | 2885916 | 2971528 | 2827335 | 2886213 | 2810196 | 2833465 |
| Total length (≥5000 bp) | 2854807 | 2913503 | 2847607 | 2765198 | 2827909 | 2898690 | 2798313 | 2828859 | 2771796 | 2812023 |
| Largest contig | 304253 | 366281 | 236830 | 399156 | 587825 | 235966 | 235908 | 171920 | 243691 | 298975 |
| GC (%) | 32,7 | 32,79 | 32,84 | 32,68 | 32,75 | 32,81 | 32,66 | 32,79 | 32,64 | 32,69 |
| N50 (bp) | 88010 | 105179 | 108064 | 124302 | 102839 | 71720 | 109371 | 91701 | 95923 | 129591 |
| N75 (bp) | 51579 | 57503 | 53272 | 69155 | 53540 | 36697 | 48970 | 40351 | 48104 | 83039 |
| L50 | 9 | 7 | 10 | 7 | 8 | 14 | 9 | 11 | 9 | 8 |
| L75 | 20 | 16 | 21 | 15 | 17 | 30 | 19 | 22 | 20 | 15 |
